# Moringa supplementation improves immunological indices and hematological abnormalities in seropositive patients receiving HAARTs

**DOI:** 10.4314/ahs.v22i2.2

**Published:** 2022-06

**Authors:** Jonah Sydney Aprioku, Ohanado Robinson, Atuboyedia Wolfe Obianime, Igbiks Tamuno

**Affiliations:** 1 Department of Experimental Pharmacology and Toxicology, Faculty of Pharmaceutical Sciences, University of Port Harcourt, Port Harcourt, Nigeria; 2 Department of Biochemistry, Faculty of Sciences, University of Port Harcourt, Port Harcourt, Nigeria; 3 Department of Pharmacology, Faculty of Basic Medical Sciences, University of Port Harcourt, Port Harcourt, Nigeria; 4 Department of Pharmacology, Faculty of Medicine, Bayero University, Kano, Nigeria

**Keywords:** Moringa, HIV, CD4+, TNF-α, immunology, HAARTs, Nigeria

## Abstract

**Background:**

Moringa oleifera Lam. is known to be of high nutritional and medicinal importance and has been demonstrated to possess a variety of biological activities.

**Objective:**

This study investigated the beneficial role of *M. oleifera* (moringa) supplementation in HIV positive subjects receiving antiretroviral drugs.

**Methods:**

Adult HIV positive individuals (104) attending the medical outpatient clinic in a tertiary health institution in Nigeria receiving highly active anti-retroviral therapies (HAARTs) were recruited in a randomized fashion for the study. Half of the subjects received moringa supplement (20 mg daily) additionally, while the others received only HAART and represented the control group. All subjects were monitored for 3 months during which their immunological status (CD4 counts and TNF-α), and hematological abnormalities at pre (baseline) and post study periods were determined.

**Results:**

Baseline levels of CD4 increased while TNF-α decreased significantly in control and moringa supplemented groups (p < 0.01). However, the post study CD4 values in the moringa group were higher and TNF-α values were lower compared to the control group (p < 0.01). In addition, baseline hematological abnormalities (anemia, thrombocytopenia, leucopenia, lymphopenia, and neutropenia) were improved but most significantly in the moringa supplemented subjects.

**Conclusion:**

The results suggest that moringa has immune-beneficial properties and improved hematological abnormalities in HIV positive individuals receiving antiretroviral therapy.

## Introduction

*Moringa oleifera* Lam (syn. *M. ptreygosperma Gaertn*), family Moringacaea, is a fast growing drought-resistant tree, indigenous to northern India^1^. Commonly called Moringa, *M. oleifera* has been known to be of high nutritional and medicinal importance^2^–^5^. The plant has been extensively studied but cannot be exhausted because of its rich pharmacological potentials. Besides having anti-inflammatory, antioxidant, anti-hyperglycemic, and hepatoprotective activities^3^,^6^,^7^, moringa has been reported to exhibit positive influence on leucocytes, lymphocytes, neutrophils, erythrocytes, hemoglobin and packed cell volume either alone^8^–^10^, or when administered in the presence of a toxicant^11^,^12^. It is widely believed that moringa supplement has positive immune modulating influence and immune enhancement property^4^,^11^,^13^,^14^. In Nigeria, leaf preparations of *M. oleifera* are widely used in folklore for the treatment of immune system related disorders, including Human Immunodeficiency Virus (HIV) infection.

HIV infection is associated with abnormalities of the immune defense system, which can affect cells in the adaptive and innate arms. The virus specifically attacks the T helper cells (or CD4 cells) and induces abnormal and dysfunctional changes in them^15^. CD4 cell destruction ultimately results in host susceptibility to opportunistic infections and eventual death^16^. HIV, which currently has no available curative drug treatment, has remained a global concern because of its devastating impact on human health and socio-economy. Presently, antiviral drug combination therapy known as the highly active antiretroviral therapies (HAARTs) are used for the disease, which effectively prevent multiplication of the virus and reduce symptoms significantly^17^. However, high disease prevalence, high cost or inadequate access to antiretroviral drugs, and unsatisfactory treatment outcomes are among other challenges with HIV treatment, especially in the developing nations^18^. Many patients are therefore compelled to develop coping strategies by seeking for alternative medications like herbal therapies. This has encouraged the use of moringa supplements by people living with HIV/AIDS, however the beneficial effect of moringa in HIV/AIDS treatment is yet to be documented. The aim of this study was therefore to investigate the effect of moringa supplement on CD4+ count, tumor necrosis factor-alpha (TNF-α) level, and existing hematological abnormalities (anemia, leucopenia, lymphopenia and neutropenia) in HIV seropositive patients receiving HAARTs. Elevated serum levels of TNF-α cause CD4 destruction and encourage HIV replication, while anti-TNF-α treatments have been shown to suppress HIV multiplication^19^. Interestingly, it has been reported earlier that moringa can reduce blood TNF-α-levels in animal models^6^,^20^. We thus hypothesize that treatment with moringa will improve hematological parameters, reduce TNF-α and increase CD4+ count in HIV positive patients.

## Methods

### Study Population

The study was conducted in the University of Port Harcourt Teaching Hospital, a tertiary health institution situated in the southern part of Nigeria. HIV seropositive patients attending medical out-patient HIV clinic in the hospital, who were on highly active antiretroviral therapies (HAARTs), were recruited into the study.

### Study Design

Demographic data of all enrolled participants were obtained using questionnaires prior to commencement of the study, which was conducted from April, 2015 to October, 2016. The study was a randomized control trial consisting of two groups. Group 1 (control) subjects received only HAART regimens, and group 2 subjects received HAART regimens and moringa supplement (Formula 10 Herbal Products, Kaduna, Nigeria; product approval number A7-0799L), given one capsule (200 mg) once daily. A total number of 104 subjects were enrolled for the study which was above the minimum sample size of 96 derived from the following equation: minimum sample size = Z × P (1-P) / E2 (Z = 1.96, i.e., 95% confidence interval; P=prevalence rate of disease = 50%; E = 0.103). Randomization was done to avoid selection bias, and a balance of participants between control group and moringa group was ensured. To be eligible for enrolment, patient must be on HAART at the time of the study, and be able to give informed consent. Patient that had any of the following conditions was not eligible and excluded from the study: (1) less than 18 years of age, (2) severely ill, (3) pregnant or intend to be pregnant within 4 months after commencement of the study, (4) has been on herbal, traditional or unorthodox medication previously, at least 2 weeks before the study, (5) on mind altering medications, and (6) terminally ill.

Participants were on the medications daily and monitored over a period of three months through evaluation of specific end points to assess their immunological status and hemato-pathologic states. For the above monitoring, blood samples were collected from participants at the beginning of the study (baseline), and after 1 month and 3 months, which corresponded to their scheduled first and second follow up visit periods, respectively in the study. The blood samples were collected separately into clean plain and EDTA tubes for measurement of CD4+ count, TNF-α concentration, as well as white blood cell (WBC), lymphocyte, neutrophil, red blood cell (RBC), platelet, and hemoglobin (Hb) levels (primary endpoints). Using the results obtained above, immunological status and hemato-pathological states were assessed (secondary endpoints).

### Analysis of CD4+, TNF-α, and Hematological Parameters (Primary Endpoints)

CD4+ estimation was performed using blood collected in EDTA tube with a Partec Cytoflow counter FMC system (Partec GmbH, Germany) which employs a ‘no lyse’, ‘no wash’ technique for counting CD4 cells^21^. Briefly, blood (50 µl) was added to 10 µl of monoclonal antibodies (Mab to CD4 receptors) and ‘no lyse’ buffer was added to the resulting mixture and incubated for 15 min. The blood sample tube was then rocked gently, attached to the Cyflow Counter, and CD4+ count value was read from the equipment in about 2 min.

Blood in the plain tube was centrifuged at 4000 rpm for 10 min and the serum was separated and stored at -80oC for TNF-α assay. TNF-α was quantified by ELISA method using UCTech's ELISA kit (UCTech, Netherland), and ELISA plate reader (BIOBASE EL10A, Bio Based Industry, Shandong, China). Briefly, a 96-well microtiter plate was coated with antibody solution (50 µl) specific for TNF-α (analyte) and filled up to 100 µl with PBS. The plate was immediately sealed to prevent evaporation and incubated at 4°C for 12 h (overnight). The antibody solution was removed by inverting the plate, and wells were washed six times with a wash buffer to remove unbound antibody. At this point, 200 µl of buffer, which served as a blocker, was pipetted into each well after which the plate was tightly sealed and kept in an incubator for 1 h at 37°C. Diluted standards (100 µl) and samples (100 µl) were added to the wells after buffer was removed. The plate was then sealed and incubated for 2 h at 37°C. Wells were washed six times, diluted detection antibody solution was added and the plates were incubated for 1 h at 37°C. Antibody solution (100 µl) was thereafter removed and the wells were washed six times again. Diluted SPP conjugate was then added to the wells and incubated for 1 h at 37°C and SPP conjugate was washed off. Finally, TMB substrate solution (100 µl) was added to the wells and allowed for 15–25 min at room temperature in the dark. The soluble blue end product produced by substrate was read at 450 nm.

Whole blood in EDTA tube was also analyzed using an automated Hematology Analyzer (Mindray 6800, China) to measure levels of the following hematological parameters: total WBC, lymphocyte, neutrophil, RBC, platelet, and Hb levels.

### Immunological Status and Hematological Abnormalities Assessments (Secondary Endpoints)

At the different time points, immunological status of subjects was classified based on CD4+ counts into: normal (>500 cells/µl), low (200–500 cells/µl), and compromised (<200 cells/µl)^22^,^23^. In addition, Hb and thrombocyte levels of participants in control and moringa groups were analyzed to identify and monitor those that have anemia or thrombocytopenia, respectively. Subjects with hemoglobin levels <12 g/dl were considered to have anemia^24^, while those that had thrombocyte counts <150 ×10^3^/µl were considered to have thrombocytopenia^25^. Leucocyte (WBC), lymphocyte, and neutrophil counts of the subjects were equally analyzed to identify and monitor those that have leucopenia, lymphopenia, and neutropenia, respectively Leucopenia was considered in subjects with WBC count <4000 cells/µl^26^, lymphopenia was defined as peripheral lymphocytes count <1500 cells/mm^3^, and neutropenia was defined as absolute neutrophils count <1500/µl^27^–^29^.

### Data Analysis

Data obtained were analyzed using SPSS version 20.0 (SPSS Inc., Chicago Illinois, USA). Descriptive statistics was used to characterize demographics and prevalence of hematological abnormalities in participants. Student's t test was used to compare between control and moringa groups. Values were considered significant at P < 0.05.

## Results

### Demographics of Participants

The age of subjects ranged between 21 and 70 years. Subjects of age bracket 31–40 years were highest in both control and moringa groups, constituting about 40%, followed by 41–50 years (about 30%), and the lowest age bracket was 51–70 years, which constituted less than 20% (Table 1). In terms of gender, women were more, and constituted about 70% (Table 1). In both groups, majority of the subjects (about 55%) had secondary education, those with university education constituted about 30%, while those without formal education were least, constituting 5%, (Table 1). Considering occupations of the subjects, self-employed was highest (about 70%), followed by civil servants (about 20%), whereas unemployed participants were least (<5%) in both groups (Table 1).

**Table 1:** Demographics of HIV-positive subjects on HAART with or without moringa supplement over a period of 3 months, showing age, sex, education, and occupation

Variable		Number (%)	
		HAART alone (Control)	HAART + Moringa
Age (year)	21–30	20 (19.2)	20 (19.2)
	31–40	40 (38.5)	42 (40.4)
	41–50	27 (26.0)	30 (28.9)
	51–70	17 (16.3)	12 (11.5)

Gender	Male	37 (35.6)	30 (28.8)
	Female	67 (64.4)	74 (71.2)

Education	No formal education	5 (4.8)	5 (4.8)
	Primary	16 (15.4)	18 (17.3)
	Secondary	56 (53.8)	54 (51.9)
	Tertiary (graduates)	10 (9.6)	11 (10.6)
	Postgraduate	17 (16.3)	16 (15.4)

Occupation	Student	5 (4.8)	8 (7.7)
	Unemployed	2 (1.9	3 (2.9)
	Farmer	2 (1.9)	8 (7.7)
	Self-employed	72 (69.2)	69 (66.4)
	Civil Servant	23 (22.1)	16 (15.4)

HIV, human immunodeficiency virus; HAART: highly active antiretroviral therapy

### Immunological Markers (CD4 and TNF-α)

Average blood CD4 counts of control subjects after the two point evaluation periods (1 and 3 months) were greater compared to the baseline value, but only the 3 months value was significant (p < 0.01), which represented 14% increase (Table 2). CD4 count of participants that received moringa (i.e., moringa group) was increased (p < 0.01) by 16 and 36% after 1 and 3 months, respectively (Table 2). When compared, the baseline CD4 counts of control and moringa groups were not significantly different from each other, but the CD4 counts of 1 and 3 months evaluation periods in the moringa group were higher (p < 0.01) than the corresponding values in control subjects (Table 2). Furthermore, baseline serum concentration of TNF-α in control subjects was significantly decreased (p < 0.01) by 24% after 1 month, and 28% after 3 months compared to the baseline value (Table 2). TNF-α was decreased (p = 0.002) in moringa supplemented subjects by 83% after 1 month and 78% after 3 months (Table 2). Intergroup comparison of TNF-α concentrations showed that there was no significant difference in the baseline values, but TNF-α concentrations in moringa subjects were lower (p < 0.01) than the control subjects (Table 2).

**Table 2:** CD4 counts and serum levels of TNF-α of HIV-positive subjects on HAART with or without moringa supplement over a period of 3 months

	HAART alone (control)			HAART + Moringa		
	
	Baseline	1 month	3 months	Baseline	1 month	3 months
CD4 (cells/µl)	425.18±257.79	440.79±180.46	482.73±187.53**	428.45±239.99	495.81±292.15**^b^	582.79±271.66**^b^
TNF-α (pg/ml)	72.47±52.66	55.34±22.90*	52.13±20.86**	74.77±41.20	12.81±10.98**^b^	16.30±3.78**^b^

Data expressed as Mean±SD, n = 104

** p < 0.01, compared with baseline

b p < 0.01, compared with control group

TNF-α, tumor necrosis factor alpha; HIV, human immunodeficiency virus; HAART, highly active antiretroviral therapy

### Hematological Parameters

Baseline WBC count of subjects in control subjects was not significantly different from the values that were obtained after 1 or 3 months (Table 3). Baseline WBC count in participants that received moringa was increased (p < 0.05) after 1 and 3 months (Table 3). Absolute count and relative concentration (percentage) of lymphocytes in control subjects did not significantly change after 1 month or 3 months compared to baseline results (Table 3), but in the moringa supplemented subjects, baseline absolute lymphocyte count significantly increased (p < 0.05;p < 0.01) after 1 month and 3 months, equivalent to 12 and 27% increases, respectively (Table 3). The percentage of lymphocytes also increased significantly by 23% after 3 months compared to the baseline value (Table 3). There was no significant change in neutrophil count over time throughout the study period in the control group (Table 3). In the moringa treated group, neutrophil count did not change after 1 month, but significantly increased (p < 0.05) after 3 months (Table 3). The percentage of neutrophils in the moringa group also increased (p < 0.05) after 3 months compared to baseline value (Table 3). The WBC, lymphocyte and neutrophil counts at the 3 months evaluation time in moringa group were significantly higher (p < 0.05) than the corresponding values that were obtained in control group (Table 3). Baseline levels of RBC and Hb in control subjects were not significantly different from the corresponding values that were obtained after 1 month or 3 months (Table 3). In the moringa treated group, baseline RBC and Hb levels were increased (p < 0.05, p < 0.01) after 3 months, and the values were significantly higher compared to the corresponding values in control group (Table 3). In addition, baseline platelet count was not significantly affected after 1 month but decreased (p < 0.05) after 3 months in control subjects (Table 3), whereas platelet count was increased after 1 month and 3 months in the moringa group (Table 3). The platelet counts in moringa group were also higher (p < 0.05) than those in control (Table 3).

**Table 3:** WBC, Lymph, Neut, platelet, RBC counts, and Hb levels in HIV-positive subjects on HAART with or without moringa supplement over a period of 3 months

Group	HAART alone (control)			HAART + Moringa		
	
	Baseline	1 month	3 months	Baseline	1 month	3 months
WBC (×10^3^/µl)	4.15±1.37	4.12±1.22	4.16±1.20	4.05±1.47	4.38±1.30*	4.41±1.39*^a^
Lymph count (cells/mm^3^)	1882.28±726.55	1738.17±714.64	1735.71±580.43	1798.31±796.20	2014.75±988.15*^a^	2275.78±995.29**^a^
Lymph (%)	45.37±9.08	42.86±10.13	42.69±9.50	44.07±10.67	45.19±13.92*^a^	54.20±26.52**^a^
Neut count (cells/mm^3^)	1797.03±646.28	1852.21±873.47	1898.14±773.05	1721.40±773.44	1780.96±706.46	2014.75±988.15*^a^
Neut (%)	42.06±8.35	44.83±11.47	44.12±12.02	42.61±10.64	40.62±10.05	48.83±15.88*^a^
Hb (g/dl)	11.64±1.79	11.43±3.70	11.84±2.69	11.97±2.19	12.02±1.65	13.15±1.33*^a^
RBC (×10^6^/ul)	4.06±0.66	3.96±1.64	3.96±1.34	3.76±0.61	3.90±1.12	4.51±0.95**^a^
Platelet (×10^3^/µl)	255.95±61.22	227.99±58.35	210.07±69.78*	238.12±64.40	250.03±63.58*^a^	258.04±91.14**^a^

Data expressed as Mean±SD, n = 104

* p < 0.05

** p < 0.01; compared with baseline

a p < 0.05; compared with control group

WBC, white blood cell; RBC, red blood cell; Lymph, lymphocyte; Neut, neutrophil; Hb, haemoglobin; HIV, human immunodeficiency virus; HAART, highly active antiretroviral therapy

### Immunological Status and Hematological Abnormalities

**Figure 1:**
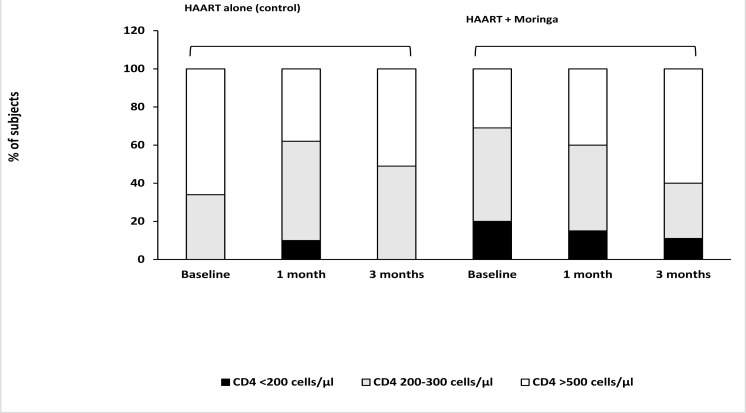
Percentage of HIV-positive subjects with CD4 levels >500 cells/µl (normal immunity), 200–300 cells/µl (low immunity) or <200 cells/µl (compromised immunity, i.e., immunocompromised) at baseline and after HAART treatment with or without moringa supplement for a period of 3 months HIV, human immunodeficiency virus; HAART, highly active antiretroviral therapy

In control group, the proportion of subjects with normal immune levels at baseline, 1 month, and 3 months were 66, 38, and 51%, respectively; those with low immune levels were 34, 52 and 49%, respectively; and those that had compromised immune levels were 0, 10 and 0%, respectively (Figure 1). In the group that received moringa supplement, 31% of subjects had normal immune levels at baseline, which increased to 40 and 60%, after 1 month and 3 months, respectively; 49% of subjects had low immune levels at baseline, which decreased to 45 and 29% after 1 month and 3 months, respectively; and 20% had compromised immune levels at baseline, which decreased to 15 and 11% after 1 month and 3 months, respectively (Figure 1).

**Figure 2:**
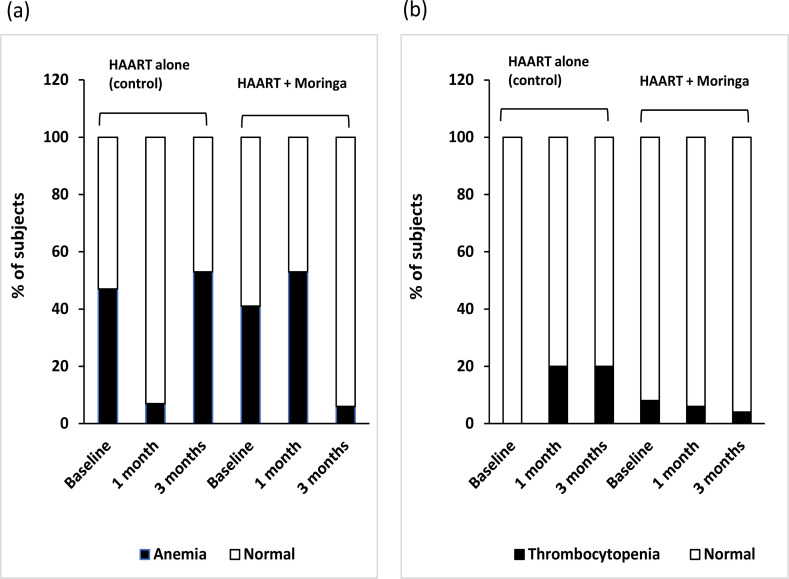
Proportion of HIV-positive subjects with (a) anemia, and (b) thrombocytopenia before (baseline) and after receiving HAART with or without moringa supplement for a period of 3 months HIV, human immunodeficiency virus; HAART, highly active antiretroviral therapy

**Figure 3:**
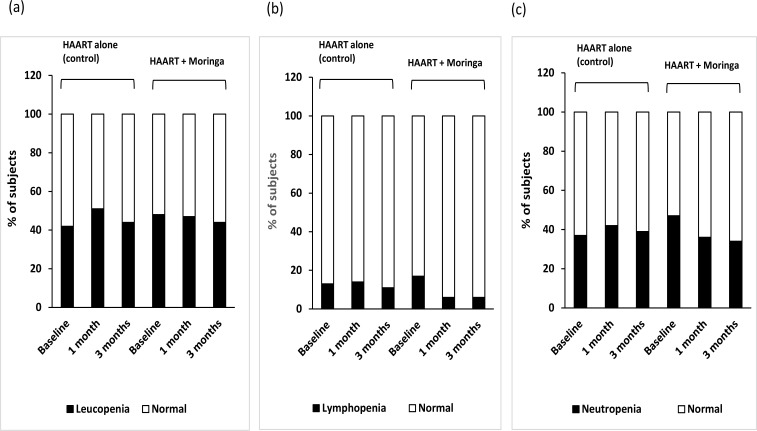
Proportion of HIV-positive subjects with (a) leucopenia, (b) lymphopenia, and (c) neutropenia before (baseline) and after receiving HAART with or without moringa supplement for a period of 3 months HIV, human immunodeficiency virus; HAART, highly active antiretroviral therapy

In addition, the population of subjects in control group with anemia was initially 47% at baseline, which dropped to 7% after 1 month, before increasing to 53% after 3 months (Figure 2a). In the moringa supplemented group, 41% had anemia at baseline, which increased to 53% after 1 month, and later decreased to 6% after 3 months (Figure 2a). None of the subjects in control group had thrombocytopenia at the beginning, but 20% had thrombocytopenia after 3 months, whereas, in the moringa group, 8% had thrombocytopenia initially, which decreased to 6 and 4% after 1 month and 3 months, respectively (Figure 2b). Furthermore, subjects in control group with leucopenia was initially 42%, which changed to 51 and 44%, while an initial 48% of subjects in moringa group with leucopenia decreased slightly to 47 and 44% after 1 month and 3 months, respectively (Figure 3a). For those that had lymphopenia, the subject populations recorded in control at the three evaluation periods were 13, 14 and 11%; and those in the moringa group were 17, 6, and 6%, respectively (Figure 3b). The results obtained for subjects with neutropenia were 37, 42 and 39%, respectively in control; and 47, 36 and 34% respectively, in moringa group (Figure 3c).

## Discussion

This study reports the beneficial effects or otherwise of moringa in HIV treatment. Levels of CD4, TNF-α and hematological abnormalities were monitored over three months in HIV positive subjects that were on HAARTs with or without moringa supplement. Out data showed that HIV infection was more in the female gender. This supported earlier reports that women are more predisposed to HIV infection than men, which has been attributed to biologic, economic, social and cultural factors^30^. Also, most of the subjects fell into the age group of 31–40 years which is similar to the results of Kandala et al.^31^ who reported that the highest prevalence of HIV infection in a Boswana population was among the 36–49 year age group. This age group represent the viable and productive section of any human population, and so justifies continuous research for improvement of HIV treatment.

Our results showed that CD4 cell counts in subjects that received moringa supplement were improved over the 3 months treatment period with significant reduction of subjects population having low CD4 counts (<300 cells/µl) and increase in subjects population with normal CD4 counts (>500 cells/µl). The immuno-beneficial effect of moringa was evidenced as the positive change in CD4 count was lesser in subjects that received HAART alone. Similar results have been reported in mice wherein moringa was shown to increase CD4 levels^32^,^33^. Additionally, TNF-α level in serum was reduced significantly in moringa treated subjects which is in agreement with results reported in similar studies in rats^20^,^34^. Diminished CD4 levels indicate poor immunity, while high concentrations of TNF-α results in negative immuno-pathological consequences^35^,^36^. Hence the improvement of CD4 and reduction of TNF-α levels by moringa in the current study demonstrates that moringa possesses beneficial immunological activity in humans. We attribute this positive immunological influence of moringa partly to its bioactive constituents like phytates, saponnins, vitamins and phenolic compounds^3^,^37^. Phytates are potent antioxidants that can enhance enzyme functions in immunological reactions, while saponnins promote production of immune mediators and also stimulate cells that function in the immune system^38^.

Hematopoiesis is usually diminished in HIV infected individuals with the consequence of reduced formation of blood cells^39^. As a result, HIV infected individuals usually present with clinical hematological abnormalities, including anemia, neutropenia, lymphopenia, and thrombocytopenia^40^. The above provide explanation for the hematological abnormalities that were observed in the HIV patients at baseline. These abnormalities may have been caused by the virus itself, antiretroviral medications or opportunistic infections. It is worthy of note that moringa treatment increased (improved) the red blood cell levels in HIV subjects, which is consistent with similar results in animal studies that reported increased red blood cell counts by moringa^41^,^42^. Hemoglobin was also increased and normalized in a large number of subjects that received moringa in the present study, causing significant improvement of baseline anemia in the subjects. This improvement was however, not seen in the subjects that received only antiretroviral drugs. This was not surprising as many of the antiretroviral drugs adversely affect hematological indices. Similarly, the subjects that received moringa showed increase in leucocyte, lymphocyte and neutrophil counts with corresponding improvement of leucopenia, lymphopenia, and neutropenia that existed before commencement of the study. The elevations in leucocytes, lymphocytes and neutrophils that were obtained are consistent with earlier reports in animal models^5^,^11^,^43^. Leucocytes are essential immunological cells, and low levels signify reduced immunity and could make HIV positive individuals susceptible to opportunistic infections. Lymphocytes and neutrophils, which are sub-populations of leucocytes, are effector cells of the innate and adaptive immune cells. This further supports the possibility of moringa having immune-modulatory potential. Furthermore, the improvement of thrombocytopenia in moringa treated participants shows that moringa may be a useful hematopoietic stimulator and suitable complimentary medication in HIV treatment. In the present study, patients were monitored for three months following HAART and moringa administration, but longer evaluation period (nine months and above) would be necessary in future studies to provide stronger evidence of the obtained results.

## Conclusion

Moringa may have immune-beneficial properties and also improve hematological abnormalities in HIV positive individuals receiving antiretroviral therapies. However, longer monitoring period of patients after moringa administration may be necessary in future studies to establish this effect.
